# Balancing acting and adapting: a qualitative study of medical students’ experiences of early clinical placement

**DOI:** 10.1186/s12909-022-03714-y

**Published:** 2022-09-03

**Authors:** Malin Sellberg, Per J. Palmgren, Riitta Möller

**Affiliations:** 1 Department of Clinical Science, Intervention and Technology, Karolinska Institutet, Stockholm, Sweden; 2 Women’s Health and Allied Health Professionals Theme, Medical Unit Occupational Therapy and Physical Therapy, Karolinska University Hospital, Stockholm, Sweden; 3 Department of Learning, Informatics, Management and Ethics, Karolinska Institutet, 171 76 Stockholm, Sweden; 4 Department of Medical Epidemiology and Biostatistics, Karolinska Institutet, Stockholm, Sweden

**Keywords:** Medical student, Undergraduate education, Supervision, Intended learning outcomes

## Abstract

**Background:**

Clinical learning experience is an important part of medical education. In the clinical learning environment, students are exposed to various aspects of medical care and may train their skills under supervision. Supervision, in which students’ learning needs and the outcomes of placements are met, is essential. The aim of this study was to explore medical students’ experiences of the early stages of clinical training.

**Methods:**

In 2021, 18 individual semi-structured interviews were conducted with medical students after their first clinical placements in semester 5. The interviews were transcribed verbatim and analyzed using qualitative content analysis according to Graneim and Lundman.

**Results:**

The findings resulted in an overall theme: *balancing acting and adapting.* Three categories described that the clinical learning environment was a big leap from campus, that personal relationships influenced learning, and that the organization of clinical placements was suboptimal. The students were encouraged to push themselves forward to practice clinical skills. This, however, did not suit all the students; the cautious ones risked becoming passive spectators. The intended learning outcomes were not frequently used; rather, the supervisors asked the students what they had learned, or the students focused on what seemed to be important on the ward. The students tried to adapt to their supervisors’ working situation and not to be a burden to them.

**Conclusions:**

Our findings show that the transition from learning on campus was sometimes abrupt, as the students had to switch to a more active learning role. Ad hoc solutions in supervision occurred, which contributed to the experience that educational responsibilities were downgraded and the opportunities for clinical training varied. Rather than trying to change the circumstances, the students opted to adapt to the busy clinical learning environment.

**Supplementary Information:**

The online version contains supplementary material available at 10.1186/s12909-022-03714-y.

## Background

In the clinical learning environment (CLE), students transform theoretical knowledge into practice in an authentic context [1, 2]. Previous research shows that the CLE has a strong influence on students’ satisfaction and achievement of the learning outcomes [3, 4]. However, the CLE is as much an educational setting as a workplace setting, where clinicians often report difficulties in balancing supervision responsibilities with clinical duties [5, 6].

Medical schools have tried to ease undergraduate medical students’ transition into the CLE [7]. In a qualitative study of students’ preparedness, Radcliffe and Lester [8] show that the most stressful transition is from basic science to apprentice doctor on the ward. In the CLE, students need to switch from learning more passively, e.g., from lectures and textbooks, to learning actively from staff and patients and applying their knowledge in a rapidly changing health care setting [7]. Thus, Prince et al. [8] propose an introduction to clinical placements, so that students know what is expected of them and so that the learning activities in the CLE would be more structured. Overall, the clinical phase of education needs more attention to better understand the factors affecting students’ experiences of the CLE and supervision in the early stages of clinical training.

The relationship between student and supervisor has been recognized as an important factor in the effective clinical supervision of medical students [9–11]. According to Kilminster et al. [12], the student-supervisor relationship begins with a discussion about the structure of the clinical training and of how and when the supervision process will be reviewed, and it continues with the identification of clear learning outcomes. In a constructive relationship, supervision has been proven to support medical students in acquiring and implementing knowledge and skills in clinical practice, but both students and supervisors confirm that, due to the conflict between clinical and educational duties, this kind of relationship may be difficult to achieve [13–15]. Remmen et al. [16] argue that educational resources are not used optimally, as much of the teaching is performed by junior doctors, and too many undergraduate students’ learning experiences are passive. These results are confirmed by Daelmans et al. [14], who describe medical students being supervised mostly by residents, who were occupied with their own duties and spent too little time on supervising students. Still, there are a limited number of studies addressing undergraduate medical students’ experiences of supervision. Further knowledge of students’ experiences could support the development of educational arrangements to better support learning.

The clinical supervisor’s responsibility is a formal assignment with an evaluative component regarding a student’s progress toward the intended learning outcomes (ILOs) [17–21]. Another important task, according to Pront et al. [22], is to individualize the supervision, since all students embark on their training with differing prior experiences upon which to build their competencies. From the supervisors’ perspective, the ILOs guide what the students need to practice to achieve them [23, 24]. Studies addressing medical students’ experiences of supervisors’ familiarity with ILOs are rare. One instrument that brings up supervisors familiarity with ILOs is the Undergraduate Clinical Education Environment Measure (UCEEM) [25]. A recent study shows that medical students rated items related to supervisors’ familiarity with ILOs lower than did students in other programs [26]. Even though there are publications on undergraduate medical education and students’ experiences of it, there is a paucity of data on the use of learning outcomes in the CLE.

In this paper, we consider students’ learning in clinical settings as a social experience wherein relationships with supervisors and health care staff are crucial. We adopt a socio-constructivist perspective on learning to explore student learning in clinical practice and use the community of practice (CoP) theory to frame the study [27]. CoP is a neo-Vygotskian theory that explains how groups of people who share a concern or passion for something learn more efficiently through regular interaction [27]. Within a CoP, peripheral members gain knowledge, acceptance, and identity and move toward the center of the community as they come to share knowledge with core members [28]. A CoP can make medical education more effective by creating a welcoming community that actively engages students in the community and by providing faculty development [29]. Thus, the overall aim of this study was to explore medical students’ experiences of the early stages of clinical training.

## Methods

### Study design

To explore students’ experiences, the authors adopted a qualitative, interpretative approach, thus examining the phenomenon in its natural setting [30]. The study was conducted within an interpretative paradigm, with the assumption that knowledge is situated, relative, and socially constructed. The findings of the study are viewed as being shaped during the interaction between the researchers and participants and do not reflect an objective truth [31]. The study adhered to the Consolidated Criteria for Reporting Qualitative Research guidelines [32].

### Context

The setting of the study was a medical university with a 5.5-year undergraduate entry medical program. The program leads to a medical degree, but doctors are not licensed until they have concluded an 18-month internship. The first 2 years mainly cover basic science education and the last 3.5 years mainly clinical education. Clinical placements are included in almost all the semesters, accounting for about 55% of the program, and are often short, with an average length of one to 2 weeks. The course in internal medicine comprises 32 weeks (48 European Credit Transfer System [ECTS]) and runs through semesters 5 (17 weeks; 25.5 ECTS) and 6 (15 weeks; 22.5 ECTS), with a break for a course entitled Scientific Methodology in Medicine (3 weeks; 4.5 ECTS) at the end of semester 5 (https://education.ec.europa.eu/education-levels/higher-education/inclusive-and-connected-higher-education/european-credit-transfer-and-accumulation-system). Internal medicine is integrated with the specialties of infectious diseases, dermatology, clinical pharmacology, geriatrics, and primary care as well as a professional development thread. It is organized into blocks with integrated theoretical teaching and clinical training. Of the four three-week–long blocks of clinical training, two take place during semester 5 and two during semester 6. During the clinical placement blocks, students rotate through various internal medicine departments as well as the emergency department. In addition, students spend 1 week (1.5 ECTS) on a geriatric ward and 2 weeks in primary care. Semester 5 starts with a three-week theory block with lectures and patient-based case seminars as well as clinical skills training. During clinical placements, the role of the supervisor is to arrange training based on the ILOs of the course and to support and develop student autonomy and independence under safe supervision. In the medical education literature, the terms *supervisor*, *preceptor*, and *mentor* are used interchangeably. In the current study, *supervisor* refers to the definition suggested by Kilminster et al. [12] that embraces the provision of monitoring, guidance, and feedback on matters of personal, professional, and educational development. For supervisors, a theoretical, web-based course in supervision is mandatory.

### Participants

Purposive sampling was employed to obtain richness and variation in the data [33]. All medical students (*n* = 177) registered on the course Scientific Methodology in Medicine in fifth semester in May 2021 were invited to participate. Information about the study was disseminated both in writing and verbally to students during the introduction of the course. Eighteen students (16 females, 2 males) with a mean age of 26.6 years (range 21–40) agreed to participate. The participants provided informed consent at the time of the interview, and they were reassured that their anonymity would be protected. Each participant received a book gift card as compensation.

### Data collection

An interview guide was constructed based on the aim of the study and our previous findings from the UCEEM questionnaire [26]. To determine the relevance and appropriateness of the questions, two pilot interviews were carried out with medical students in the fifth semester in the spring term of 2021. No modifications to the interview process or interview guide content were necessary. All the interviews, carried out by the first author (MS) in June 2021, were conducted face to face using the Zoom platform and were audio- and video-recorded. The interviewer took field notes during the interviews, which constituted the units of analysis and lasted from 30 to 70 minutes.

### Data analysis

The collected data were analyzed by inductive qualitative content analysis with a constant iterative comparison coding to explore the data [34, 35]. During the process of transcribing, a diary was used to collect immediate reflections. Interpretations were made to latent and manifest content analysis [34, 35]. MS anonymized and transcribed the interviews verbatim, identified meaning units, and coded the text entered into the NVivo software package [36]. All the transcribed interviews were read by all the authors. The analysis was continually discussed within the research group using an iterative process until consensus was reached [34, 35, 37]. The analysis followed the steps described by Graneheim and Lundman for qualitative content analysis [35]. As a final analysis step, the authors interpreted the underlying latent content of the data [38].

### Trustworthiness

To ensure trustworthiness, the criteria of Lincoln and Guba were applied [39]. Several strategies to ensure credibility were used: prolonged engagement with the data, investigator triangulation, and persistent observation. To enable readers to assess the transferability of the findings, thick descriptions of the context, participants, and research process were provided. To achieve dependability, the standards for qualitative content analysis according to Graneheim and Lundman were applied [35]. To achieve transparency and, hence, confirmability, rich quotations were provided. Reflexivity was carefully considered. MS, who conducted the interviews, was a former adjunct of the physiotherapy program and a PhD student at the time of the study, with no personal relation to the students. PP was full-time researcher and senior educationalist. RM was a medical doctor, part-time clinician, part-time researcher, and the director of the course in Scientific Methodology in Medicine at the time of the study. The interviewer took notes during the interviews, which were discussed within the research group. The research team held recurrent discussions regarding preconceived assumptions in the process of collecting, analyzing, and interpreting the data.

## Results

Based on the interviews, the analysis resulted in one overarching theme—*balancing acting and adapting*—and three manifest categories: *the clinical learning environment*—*a big leap from the campus*, *personal relationships influenced learning*, and *suboptimal organization of clinical placements* (Table 1). The categories are presented using eight subcategories illustrated with supporting quotations.

**Table 1:** Overview of the identified subcategories, categories, and the theme

Subcategories	Categories	Theme
Transition in the role of the learner	The clinical learning environment—a big leap from the campus	Balancing acting and adapting
Intended learning outcomes were hidden and vague		
Ambivalence toward peers	Personal relationships influenced learning	
Supervisors as role models: two sides of a coin		
Becoming an acknowledged member of the team		
Avoiding criticism		
Ad hoc solutions when supervisors were absent	Suboptimal organization of clinical placements	
Educational responsibilities were downgraded		

### Balancing acting and adapting

The CLE differed from student expectations. The course management encouraged the students to push themselves forward to act at the clinic, which did not suit everyone. The CLE was considered beneficial to students who were able to push themselves forward whereas cautious students risked becoming passive spectators. The ILOs were not regularly used in the clinic; instead, the supervisors asked what the students had learned prior to the clinical training, and the students focused on what they considered important to learn in the placement. Supervision without a focus on the learning outcomes, together with short placements, made it difficult for the students to understand their roles and affected their feelings of being prepared for future work. The students tried to adapt to the environment without being a burden on their strained supervisors, whom they considered stressed in a difficult work situation.

### The clinical learning environment—a big leap from the campus

This category was based on two subcategories: *transition in the role of the learner* and *intended learning outcomes were vague and hidden.*

#### Transition in the role of the learner

The medical students communicated that the transition from the campus-based learning environment to learning in a stressful clinical environment was abrupt and cumbersome. They described it as moving from an environment where they had been recipients of information to an environment in which the course leaders and more senior students encouraged them to take the initiative to obtain opportunities to practice clinical skills. The conditions for learning were perceived as tough and favoring students who were able to push themselves forward while they made others feel insecure and reconsider their career choices. The students felt that the number of learning occasions was not sufficient and that opportunities to follow up and practice certain activities again were few. Nor was it solely up to the students to decide what to do in the clinic; it was decided together with the supervisor whether students’ initiatives were suitable. If an initiative was not accepted by the supervisor, some students persisted until they succeeded and were allowed to do what they wanted while some students found other activities to do on their own. The students said that shy students, those without the capability of pushing themselves forward, and those who chose not to use this manner risked completing the clinical course merely as passive spectators.

*In the end, it’s what you make of it. You really have to push. If you want to do things, you have to ask about it, and you have to be a little assertive. If you do not ask for something, you can’t do anything. It is very important that you take initiative for yourself. If you just wanted to stay at the side all the six weeks, you could have done it and not achieve so much.* Student 9.

The students revealed that how prepared they felt to take the initiative and handle patients on their own varied. There was also variation in how quickly they adapted to the new circumstances. The necessity of taking the initiative from day one resulted in psychological stress for some students, and students who viewed themselves as people oriented considered that there was a risk of loss of confidence in this kind of learning environment. The students stated that it would have been easier if they had received a couple of days to familiarize themselves with the context before taking initiative on their own. Volunteering to perform a clinical procedure that they had seldom or never practiced was experienced as jeopardizing patient safety.

*They said that we should throw ourselves out, take patients ourselves immediately. But some need a longer time to get used to the environment. I do not think that palpitations or stress generate anything good for us. I do not think we will become better doctors or become competent faster when we are thrown out. The medical students who are people oriented and want to think a little and not push their way forward without regard for others, it is possible to break them down. I think it’s easy to lose individuals who would have become very good doctors. If I got the possibility to get used to the situation for a day or two, I would have done better and felt better and had better self-confidence and learned more.* Student 15.

The students stated that the CLE was more overwhelming and challenging than they had expected. The workplace was perceived as unstructured and opportunistic. The students were surprised by how much knowledge the supervisors had to keep in mind. It was difficult for them to contextualize theoretical knowledge to understand the supervisors’ quick decisions and clinical reasoning and to comprehend what was of importance for the patient. The students also expressed negative impressions of the number of administrative duties and the scarcity of the time the clinicians spent with patients.

*That you spend so much time in front of the computer. That was what surprised and disappointed me the most.* Student 10.

#### Intended learning outcomes were hidden and vague

Students who prepared themselves for clinical placements by reading the ILOs beforehand stopped doing so, since the supervisors did not seem to be aware of them or use them. The students also remarked that there were too many learning outcomes to keep track of and that they were ambiguous and difficult to understand.

*There are many intended learning outcomes to begin with. They can be quite vague. You can interpret them in many different ways, and sometimes it is quite difficult to know concretely what is important to learn and become better at.* Student 15.

The students told the interviewer that, in placements they considered better, the supervisor initiated dialogue with them about the ILOs, students’ previous experience, and their learning needs. When the ILOs were clear and coherent, it was easier for the students to focus on learning activities and achieve the outcomes. Conversely, when the supervisors did not discuss or illuminate the ILOs for the placement, the students focused instead on engaging in learning based on what they thought most important to know at the placement.

*I did not attempt to fulfill the intended learning outcomes; I rather tried to discern what appeared to be important at the ward.* Student 6.

Some supervisors asked the students which semester they were in and what subjects they had studied recently and, at best, adapted their teaching accordingly. Some supervisors focused on teaching and learning activities that they had employed with previous students or on what they considered relevant to know at the placement. When supervision entailed only observing the supervisor’s patient encounter, the students considered the encounter as contributing to their learning to a limited extent.

*They checked what kind of theory blocks we have had or asked, “What did you do during the last placement? What was included in your most recent exam?” A good supervisor tried to adapt their teaching to what we had studied recently. At a less optimal placement, the supervisor did not care about that and let us take any patients that we did not know much about. In such a case, I just met a patient, not much more; there was no teaching around it.* Student 2.

Another aspect that influenced the students’ possibilities of accomplishing the ILOs was the length of placements. During a placement of one week, it was more difficult to find patients suitable for them to hone their clinical skills with.

*There were quite a few patients the week I was placed there, so, I had to train my ability to take patient history, to do physical examination, and train my communication skills and writing patient record notes, all on the same patient. There was no opportunity to do certain things because that patient group did not exist.* Student 5.

In the students’ experience, without clear learning outcomes, they were unsure whether they had learned sufficiently during the placement. The most satisfying feeling was achieved when they felt that they had learned a great deal, regardless of the ILOs. However, the absence of learning outcomes created uncertainty regarding how well prepared they were for future work as medical doctors.

*It is very unclear. I still feel very insecure about how well prepared I am for my future job. I feel that all the time. Did I really get everything I need now from this course for the future work? No, I do not know that, is the answer to this question.* Student 16.

### Personal relationships influenced learning

This category was based on four subcategories: *ambivalence toward peers*, *supervisors as role models: two sides of a coin, becoming an acknowledged member of the team,* and *avoiding criticism*.

#### Ambivalence toward peers

The students said that having peers at the same placement facilitated their learning, since two students dared to take on tasks that they would not have attempted on their own. Another advantage with peers was that the students could provide feedback to each other or discuss the feedback they received from their supervisor, or other issues, without time constraints. Having a peer at the same placement also created opportunities to join in a learning activity initiated by the other student’s supervisor.

*When we are several students at the same ward, the other students’ supervisors can suggest, “We thought we would go and do this, do you want to come along?”* Student 7.

A common lunchroom for all the students provided a physical, undisturbed space to share experiences, exchange information, ask questions, and support one another. In particular, when students had difficult experiences with patients or supervisors, peer support was experienced as important and enabled them to better cope and continue with the course.

*When it was tough and I doubted myself, it was the talking to other students that helped me to get through. It made a big difference if somebody else had been at the same placement.* Student 8.

An additional way to obtain help was to communicate with more senior students through social media. In such forums, senior students shared their experiences and openly responded to questions regarding clinical placements. They gave practical advice, for example, considering a specific placement.

*Somebody had compiled a list of “10 things you should think of before you start your clinical placement.” Even if it comprised easy actions such as “Arrive on time” or “Say hello to everybody,” “Make sure your badge for the computer works,” “You can get help with this …*” *There were quite a few questions to the senior students* [on the social media]*, which they answered. That was helpful.* Student 17.

However, having peers at the same placement was not always an advantage. The students perceived that it could result in diminished attention paid by the supervisor to each student. Someone who did not behave as expected or perform well was someone the students did not want to be associated with. Also, a peer who did not take initiative or perform well was considered a burden on other students.

*If you are together with a pushy peer, you can practice clinical examination together, discuss, and encourage each other to push the supervisor: “We can do this,” and I feel safe when we are together” But if it is a peer who reduces the quality of my clinical education, it is a disadvantage.* Student 3.

#### Supervisors as role models: two sides of a coin

Supervisors became in many ways role models for the students, in how they acted both professionally as medical doctors and as supervisors and facilitators of learning. They could become either someone the students looked up to or, on the contrary, someone they distanced themselves from.

*You learn quickly what kind of person you do not to want to be as a doctor. There are people you meet and think, “I want to be more like this person,” but we also remember the negative experiences: “This is not the way I will act if I have students in the future; I do not want the students to sit and twiddle their thumbs and have nothing to do and feel that there is no place for them.”* Student 14.

The students expressed how supervisors who made a bad impression prompted them to question their choice of profession. They also stated that they lacked confidence in this kind of supervisor, and they felt that their possibility of engaging in clinical activities was diminished. Further, supervisors exhibiting disrespectful behavior were challenging for the students to handle.

*It was difficult to deal with, much more difficult than being near dying patients, to see a chief physician who does not respect patients and is not nice to the colleagues. How can I find my place there, and how can I handle it? I think I will take it with me in my future professional practice; I think it will affect how I will think and how I think I should not behave.* Student 10.

#### Becoming an acknowledged member of the team

The students appreciated someone greeting and welcoming them upon arrival on the first day and coworkers learning their names. Additionally, if they were assigned a task, they experienced a sense of belonging with the staff. They also described how important it was to be looked upon as an asset for their supervisors rather than being perceived as a burden. When students were assigned authentic clinical tasks, it was easier for them to participate in discussions with the staff, and they felt more like members of a team.

*It is valuable that we sometimes get opportunity to discuss, that I can say things. I experienced during the rounds that, when I got to introduce my patient and the nurses reported to me about my patient, then I felt very involved and a part of the team.* Student 10.

The length of the placement also influenced student experiences of being a member of a team. They perceived that it takes time to become a team member, and to be able to do so during a placement as short as 1 week was considered unlikely. The students noted that the first days of a placement were largely spent trying to comprehend the organization, the role of the coworkers at the placement, and the kinds of patients cared for at the placement. Conversely, longer placements abetted the sense of becoming a team member.

*During the first days, you try to understand the routines, you start to know the staff, and you do not spend that much time with the patients. During the second week, I met quite a lot of patients for myself and was confident with it. I knew what was going on, and I knew how the rounds were organized. Two weeks at the same placement means so much more for us; it is more than twice as much as a week.* Student 15.

The students noticed other obstacles to becoming acknowledged team members. When the supervisor did not meet them on the first day of the placement, or if they were not introduced to the staff or to the duties on the ward, it felt as though they were on a study visit. Without a proper introduction to the placement, the students experienced a feeling of not being included in the team.

*It would have been great if the supervisor would have told the staff at the placement that I was a medical student. Then it would have been easier; I did not really know what the rest of the staff were doing. Then, it would have been easier to follow their work.* Student 18.

The students remarked on large staff rotations that also included their supervisors. This constant movement during their placements also contributed to the difficulty in becoming a team member.

*There was a steady flow of staff; they came and went every day, and therefore it gets harder* [to become part of the team]*.* Student 5.

#### Avoiding criticism

The students were careful in their criticism concerning negative experiences. They viewed their supervisors as potential employers. If they were critical, this would reduce the possibility of returning for an internship at the same hospital. Instead, they wanted to show their best side to the staff in the ward to increase their chances of being offered a job. It meant that they continually chose not to comment on supervision they considered poor in order not to appear cumbersome.

*All the people I meet can possibly become my employers. So, it is an incredible balance of power that you must deal with all the time. I must be friendly and skillful but not annoying. I do not want to change from that supervisor to another, because then he will know that I did not like him, and it will be hard for me in the future. I still want to be liked here, because I might want a job here.* Student 3.

Some students viewed the placements as hierarchical and regarded the interns as focusing on their own contribution as doctors, since they were continuously judged by a colleague in a higher position. The hierarchical attitude further contributed to refraining from criticism. The undergraduate students remarked that the interns took the focus from them, and the students considered themselves as standing lowest in rank.

*I am a medical student, and, as a doctor, I have the role of authority. But as a student I am lowest in rank, which makes you a bit insecure. I feel like an apprentice. I am at the bottom of the food chain.* Student 6.

### Suboptimal organization of clinical placements

This category was based on two subcategories: ad hoc *solutions when supervisors were absent* and *educational responsibilities were downgraded.*

#### Ad hoc solutions when supervisors were absent

The students described that they were not always met by a supervisor who was expecting their arrival. Thus, they had to start the placement by seeking their supervisors or by looking for someone else to supervise them. This resulted in ad hoc solutions in front of the students, with the staff discussing who should act as supervisor. A common situation was that the person who would be a supervisor got the information from the whiteboard.

*The supervisor probably does not know that you would be here, but they will see it on the whiteboard, where it is written, “Plus student.”* Student 3.

Some of the circumstances in which no supervisor was assigned to the students at their arrival were unpredictable, e.g., sick leave. According to the students, no backup plan seemed to be available on those occasions. At other times, no supervisor was available due to schedule changes for the intended supervisors. The appointed supervisor may have also been assigned new duties that were not compatible with the supervision.

*It was unclear, when I got there; the person who would have been my supervisor did not work that day and the other possible supervisor was on call, so I would not go with her either. I looked her up first, and she said, “It’s probably best that we find someone else.” I ended up having to follow one of the senior doctors. And he was also a bit, “Well, yes, yes, okay, it will be okay.”* Student 18.

#### Educational responsibilities were downgraded

The students experienced that the educational duties were not prioritized. The supervisors prioritized clinical duties with patients over supervision. The students said that some supervisors were aware that they ignored them and their supervisory obligations due to stress.

*When we were going to do a physical examination, she just came in and took over. She thought that the examination took too long a time for us. I think she was very aware of what she was doing but that she was stressed.* Student 4.

Student initiatives to engage in the clinical work could be met with resistance by supervisors due to time constraints or unwillingness to supervise. Not everyone who became a supervisor was satisfied with their assigned task of supervision.

*I said I could write patient record notes, but the supervisor said, “No, it takes such a long time, and I still have to proofread them.” Even if I tried, they had no will or time to supervise.* Student 17.

Several supervisors were residents who prioritized their own learning. They were described as inexperienced and unfamiliar with the routines on the ward, indicating that they struggled to understand their own clinical tasks at the placement. Senior supervisors were considered better at combining supervision simultaneously with clinical work.

*The supervisor was doing his general residency; he could not handle it. He tried to understand what he was doing and did not know what would happen during the day. He was continuously given new tasks and informed about what was expected of him. It affected my learning. More experienced supervisors handle supervising and working clinically at the same time.* Student 14.

The supervisors made the right choice by prioritizing the patients, according to the students. To ease their supervisors’ burden, students put their own learning needs aside and refrained from active participation. Consequently, the students took on a passive role and observed what was happening in the placement.

*I took my responsibility from an educational perspective, but I also had to take my responsibility from a social perspective. Should I prioritize my learning or that this resident should be able to do his job today? It was something you had to handle all the time. You push* [your] *own learning aside and try to learn by observing instead of asking or discussing. I took the social responsibility in those situations.* Student 3.

## Discussion

This study aimed to explore third-year medical students’ experiences of their early stages of clinical training. The interviews showed that the transition from the campus-based learning environment to learning in CLE was sometimes abrupt. The senior students and course leaders encouraged students to take initiative and push themselves forward to be able to train clinical skills. This attitude did not suit all the students, nor was it solely up to the students to decide what to do in the clinic. The ILOs were not in focus; rather, the supervisors asked which placements the students had had most recently, or the students focused on what seemed most important at the placement. Due to absent supervisors, clinical teaching was organized by ad hoc solutions. To become an acknowledged member of the teams at the placements was considered important.

Malau-Aduli et al. [40], who studied medical students in their first clinical year, report that students were anxious about the transition from campus-based learning to the CLE, which included several new and confusing learning situations. Further, Atherley et al. [41], who conducted a scoping review on the same topic, found that students were stressed by frequent changes in the context. Our findings are in line with these studies. Indeed, the students experienced the conditions for learning in the CLE as being tough and favoring students who were able to push themselves forward while others felt insecure and even reconsidered their career choices. Providing students with authentic and meaningful learning opportunities embedded in the clinical practice is an essential part of medical education [42]. O’Brien et al. [43] suggest that exposure to the routines and norms that students encounter in clinical settings would make the process easier. By contrast, the same authors argue that, instead of considering students’ struggles as representing inadequate preparation for the transition to the CLE, they should be framed as a transformative learning process [43]. However, the transition phase seems overlooked, and these findings together indicate a need for further research, not only from students’ but also from the supervisors’ and faculty’s perspectives.

In outcome-based education, ILOs indicate what the students are supposed to know and the competencies they are expected to have developed after the completion of a certain course [44]. In our study, the students considered the ILOs to be too many and difficult to understand. Also, the supervisors’ familiarity with the outcomes were experienced as relatively low, resulting in unclear goal fulfillment and uncertainty among the students as to whether they were prepared enough for future duties as medical doctors. Weissberg [45] describes that statements of ILOs often use abstract terminology to frame higher-order cognitive competencies, such as critical analysis and problem-solving. Further, Sadler [46] notes that these terms may be broadly interpreted and that adding more words to learning outcomes most likely does not solve this problem. As the students described that there were spontaneous and unplanned changes among supervisors, it may not be realistic to find a supervisor who is familiar with all the ILOs. However, by not having clear ILOs as a navigation tool to guide both students and supervisors toward the desired result—the success of the students—the course and the curriculum will stay murky.

To understand students’ learning in the CLE, it may be valuable to theoretically approach the process of becoming a medical doctor with the support of Wenger’s theory of CoP [47]. In a broad sense, medical training can be thought of as a trajectory leading to full participation within the medical profession CoP [27, 48, 49]. The students are temporarily placed in clinical settings, where they, through increased participation over time, move from the periphery toward the core of the CoP [27, 28]. The students in the present study confirmed that this process takes time and that, without being welcomed and introduced to the staff and duties in the beginning of the short placements, they never became legitimate practitioners. These findings support the work of Egan and Jaye [47], who describe that students in hospitals or health service settings easily remain at the periphery of the community as transient members who are granted only legitimate peripheral participation. An introduction to the placement, longer placements, and authentic tasks were factors that the students in the current study believed enabled them to become legitimate members and move from the outside to the inside of the CoP. Without these “door openers” into the CoP, the students have difficulties to get enough legitimacy to be treated as potential members and consequently risk to became passive spectators.

The students in the present study experienced that the educational responsibilities concerning clinical supervision were downgraded in the clinic. In a review to understand the enablers of and barriers to effective clinical supervision, Rothwell et al. [18] found that, often, supervision was not a priority in a clinic and that the key factors hindering effective clinical supervision were lack of time and a heavy workload. Further, the review shows that, with a lack of commitment from organizations and managers, it is unlikely that the importance of supervisors will be embedded in the organizational culture. Instead, the leadership acts as a barrier to successful clinical supervision, as the clinical duties must be prioritized [18]. Rather than continuing to embrace a model in which students need to have a pushy attitude to be able to learn clinical skills, we suggest that future studies address the clinical organization of supervision and devise a system for establishing a favorable organization that integrates the needs of patient care and clinical education.

The results of this study are based on rich findings from interviews and a clear theoretical perspective. To increase the transferability of the findings to other universities, a careful description of the context was provided as well as rich quotations from students. It was considered a strength that two researchers had experiences from other programs and could contribute outsiders’ perspectives. The credibility may be questioned, as our data were derived from individual interviews, which may be interpreted in various ways. The collected data were considered rich, and the findings confirm and add information regarding medical students rating of their CLE [26]. One potential limitation was that RM was a director of the course from which the students were recruited, which consequently may have affected the students’ willingness to make a good impression by volunteering to participate or may have discouraged them from speaking freely. However, the students had no relation to the first author, who organized, conducted, and anonymized the interviews. Consequently, any power imbalances in the data collection were reduced. Using a virtual platform for data collection was necessary due to COVID-19 restrictions. We argue that it was beneficial, as the students got the chance to choose the physical place for the interview, with reduced preparation time and travel costs [50, 51]. Additionally, during transcription, it was advantageous to return to the interviews and interpret subtle nuances in tone and body language [50].

We focused here on medical students’ experiences of the CLE during their first longer clinical placement by using qualitative, semi-structured interviews as the data source. Future studies should use different data sources, e.g., clinical supervisors, clinical leadership, and medical students from later semesters, to obtain a further understanding of medical students’ CLE and different phases in their clinical training and to include the perspective of the clinical organizations. It would also be useful to use other data collection methods, e.g., questionnaires focusing on students’ knowledge of the ILOs for other courses. Also, a study among supervisors and faculty on how to prepare students for the transition to the CLE would be interesting.

## Conclusions

The interviews showed that the transition from learning on campus was sometimes abrupt, as the students had to switch to a more active learning role. Course leaders and senior students encouraged the students to act and push themselves forward in the clinic, which did not suit everyone. Ad hoc solutions in supervision occurred, which contributed to the experience that educational responsibilities were downgraded and gave students varying possibilities of clinical training. Rather than trying to change the circumstances, the students opted to adapt to the busy CLE.

## Supplementary Information

**Additional file 1: Appendix 1.** The Interview guide.

## Data Availability

The datasets generated and/or analyzed during the current study are available from the corresponding author on reasonable request.

## Abbreviations

CLE: Clinical learning environment
CoP: Communities of Practice
ILOs: Intended Learning Outcomes
UCEEM: Undergraduate Clinical Education Environment Measure
