# Supplementary material for: Balancing acting and adapting: a qualitative study of medical students’ experiences of early clinical placement
Source: BMC Med Educ. 2022 Sep 3;22:659. doi: 10.1186/s12909-022-03714-y (PMC9440768; doi:10.1186/s12909-022-03714-y)
Supplement: Supplementary file 1 — Additional file 1: Appendix 1. The Interview guide. [file 12909_2022_3714_MOESM1_ESM.docx]

**Appendix 1.** The Interview guide

| - **From campus to the clinical learning**   Can you please describe what it was like to go from campus-based teaching and learning, to teaching and learning in a clinical environment? |
| --- |
| - **Introduction**   Can you please describe your first meeting with your clinical supervisor? |
| - **Intended learning outcomes**   Can you please tell me about your experience whether the supervisor knew about the intended learning outcomes? |
| - **Impacting on learning**   Do you feel that you have been able to influence the learning circumstances during your placement? |
| - **Closure**   Is there anything else you would like to add that we have not been addressed that concerns your experience of introduction to your clinical placement or intended learning outcomes? |
